# Host and nonhost bacteria support bacteriophage dissemination along mycelia and abiotic dispersal networks

**DOI:** 10.1093/femsml/uqae004

**Published:** 2024-02-20

**Authors:** Claire Périat, Thierry Kuhn, Matteo Buffi, Andrea Corona-Ramirez, Mathilda Fatton, Guillaume Cailleau, Patrick S Chain, Claire E Stanley, Lukas Y Wick, Saskia Bindschedler, Diego Gonzalez, Xiang-Yi Li Richter, Pilar Junier

**Affiliations:** Laboratory of Microbiology, University of Neuchâtel, Rue Emile-Argand 11, 2000 Neuchâtel, Switzerland; Laboratory of Microbiology, University of Neuchâtel, Rue Emile-Argand 11, 2000 Neuchâtel, Switzerland; Laboratory of Eco-Ethology, University of Neuchâtel, Rue Emile-Argand 11, 2000 Neuchâtel, Switzerland; Laboratory of Microbiology, University of Neuchâtel, Rue Emile-Argand 11, 2000 Neuchâtel, Switzerland; Laboratory of Microbiology, University of Neuchâtel, Rue Emile-Argand 11, 2000 Neuchâtel, Switzerland; Laboratory of Microbiology, University of Neuchâtel, Rue Emile-Argand 11, 2000 Neuchâtel, Switzerland; Laboratory of Microbiology, University of Neuchâtel, Rue Emile-Argand 11, 2000 Neuchâtel, Switzerland; Los Alamos National Laboratory, Bioscience Division, P.O. Box 1663, NM 87545, Los Alamos, United States; Department of Bioengineering, Imperial College London, B304, Bessemer Building, South Kensington Campus, SW7 2AZ, London, United Kingdom; Helmholtz Centre for Environmental Research UFZ, Permoserstrasse 15, 04318, Leipzig, Germany; Laboratory of Microbiology, University of Neuchâtel, Rue Emile-Argand 11, 2000 Neuchâtel, Switzerland; Laboratory of Microbiology, University of Neuchâtel, Rue Emile-Argand 11, 2000 Neuchâtel, Switzerland; Laboratory of Microbiology, University of Neuchâtel, Rue Emile-Argand 11, 2000 Neuchâtel, Switzerland; Laboratory of Eco-Ethology, University of Neuchâtel, Rue Emile-Argand 11, 2000 Neuchâtel, Switzerland; Institute of Ecology and Evolution, University of Bern, Baltzerstrasse 6, 3012 Bern, Switzerland; Laboratory of Microbiology, University of Neuchâtel, Rue Emile-Argand 11, 2000 Neuchâtel, Switzerland

**Keywords:** bacterial–fungal–bacteriophage interactions, infection, multiplication, fungal highways

## Abstract

Bacteriophages play a crucial role in shaping bacterial communities, yet the mechanisms by which nonmotile bacteriophages interact with their hosts remain poorly understood. This knowledge gap is especially pronounced in structured environments like soil, where spatial constraints and air-filled zones hinder aqueous diffusion. In soil, hyphae of filamentous microorganisms form a network of ‘fungal highways’ (FHs) that facilitate the dispersal of other microorganisms. We propose that FHs also promote bacteriophage dissemination. Viral particles can diffuse in liquid films surrounding hyphae or be transported by infectable (host) or uninfectable (nonhost) bacterial carriers coexisting on FH networks. To test this, two bacteriophages that infect *Pseudomonas putida* DSM291 (host) but not KT2440 (nonhost) were used. In the absence of carriers, bacteriophages showed limited diffusion on 3D-printed abiotic networks, but diffusion was significantly improved in *Pythium ultimum*-formed FHs when the number of connecting hyphae exceeded 20. Transport by both host and nonhost carriers enhanced bacteriophage dissemination. Host carriers were five times more effective in transporting bacteriophages, particularly in FHs with over 30 connecting hyphae. This study enhances our understanding of bacteriophage dissemination in nonsaturated environments like soils, highlighting the importance of biotic networks and bacterial hosts in facilitating this process.

## Teaser

Demonstration of the important positive role of host carriers and fungal highways in the dissemination of bacteriophages in soil.

## Introduction

Bacteriophages are viruses able to infect and replicate within bacteria (Rohwer et al. 2009). These viruses affect the competition dynamics, structure, and evolution of bacterial populations (Weitz and Wilhelm 2012, Middelboe and Brussaard 2017). The coevolution between bacteriophages and their bacterial hosts is considered an important driver for the vast phenotypic and genotypic diversity found in bacterial populations (Koskella and Brockhurst 2014). Moreover, bacteriophages are known to alter competition between bacterial strains (Koskella et al. 2012, You et al. 2022), promote biodiversity (Buckling and Rainey 2002), and mediate horizontal gene transfer (Canchaya et al. 2003, Paul 2008). Given the abundance of bacteriophages in nature and their potential impact on the selection and evolution of their host, bacteriophage research has gained attention in microbial ecology (Koskella and Brockhurst 2014). One of the most significant bottlenecks to understanding the importance of bacteriophages on the evolutionary dynamics of bacterial populations is the mechanisms governing the encounter of bacteriophages and their host. Unlike many of their host bacteria that express active mobility, bacteriophages are thought to encounter suitable hosts mainly by diffusion (Diaz-Munoz and Koskella 2014, Simmons et al. 2018). The typical diffusion constant of a viral particle has been reported to be in the order of ca. 0.5–1.0 × 10^−7^ cm^2^ s^−1^, suggesting that the diffusion of bacteriophages by Brownian motion at the nanoscale is limited (Dennehy 2014). This limitation is particularly relevant in environments such as soils, where waterborne diffusion is already highly restricted. In soils, viral communities have been shown to exhibit spatial structuring, thus hinting at the role of dispersal limitation for community assembly (Santos-Medellin et al. 2022).

Dispersal limitation in soils results from the high density of porous spaces with varying degrees of water saturation, the diversity of solid surfaces with varied physicochemical properties, and the intermixing of solid–liquid–gaseous phases (Crawford et al. 2005, Or et al. 2007). Water saturation is a determining factor in the composition of bacterial communities, as it affects the diffusion and bioavailability of nutrients (Crawford et al. 2005), as well as the dispersal of bacteria themselves (Kohlmeier et al. 2005, Or et al. 2007). Moreover, the soils’ physical structure (i.e. connectivity, the distribution of water and nutrients) determines the access to resources by coexisting or competing populations of microorganisms (Erktan et al. 2020, Dubey et al. 2021). Variation in the connectivity of the soil pores and the water films results in the creation of refuges impacting the probability that consumers/predators encounter food sources/hosts (Erktan et al. 2020). In this context, the hyphae of filamentous fungi provide indispensable opportunities for encounters and interactions between other members of the soil microbiota (Bielcik et al. 2019). The mycelium of fungi and oomycetes have been shown to effectively connect air-filled water-unsaturated soil pores (Kohlmeier et al. 2005, Wick et al. 2007). The development of continuous liquid films along hyphae provides a network for the dispersal of bacteria, called fungal highways (FHs) (Kohlmeier et al. 2005).

The biotic networks created by FHs could contribute to or hinder the movement of bacteriophages from host-poor to host-rich areas in fragmented soil habitats. For instance, previous studies indicated that bacteriophage diffusion along mycelia is limited in unsaturated environments (You et al. 2022). Under water-saturated conditions, mycelia have been shown to promote bacteriophage retention depending on the physicochemical properties of the bacteriophage and the mycelium (Ghanem et al. 2019). Nevertheless, transport of bacteriophages along FHs has been observed with the help of hyphal-riding bacteria acting as carriers (You et al. 2022). You et al. observed that *Escherichia* T4 virus reversibly attaches to a motile nonhost bacterium (*Pseudomonas putida* KT2440), and uses it as a carrier to ride across hyphae of the oomycete *Pythium ultimum* to reach its host (i.e. *Escherichia coli*). In contrast, in the absence of carrier bacteria, the bacteriophage was not detected across the unsaturated area connected by the hyphae (You et al. 2022). This facilitated transport of bacteriophages by riding on nonhost bacteria was also shown to take place in biofilms (Yu et al. 2021) and in soils (You et al. 2022). Together, these studies highlight the importance of bacterial carriers on bacteriophage dissemination, resulting from the combined effect of diffusion and transport in the absence of reproduction in a nonhost carrier. In contrast, unassisted dissemination of bacteriophages on FHs by diffusion has been shown to be negligible.

While prior studies used nonhost carriers to demonstrate bacteriophage transport along FHs, the impact of host carriers and the possibility of bacteriophage replication within the host during dissemination has not yet been investigated. Our study bridges this knowledge gap by addressing the following questions: (1) to which degree do bacteriophages diffuse unassisted within liquid films established on the surface of abiotic or biotic (e.g. FHs) dispersal networks? (2) What is the impact of host bacterial carriers compared with nonhost carriers on the rate of bacteriophage dissemination along biotic and abiotic networks? This study demonstrates the combined positive effect of transport and multiplication in the hosts carrier for the dissemination of bacteriophages in structured environments connected via biotic networks.

## Materials and methods

### Microorganisms and culture conditions

The experiments were conducted with the soil bacterium *P. putida* (Benedetti et al. 2016). Two motile strains of this species, strains DSM291 and KT2440, were used as host and nonhost carrier, respectively. The noncompatibility of the second strain with the two bacteriophages was controlled beforehand via lysate plate assay. Strain DSM291 was purchased from the German Collection of Microorganisms and Cell Cultures (DSMZ). Bacterial cultures were performed on standard nutrient agar or nutrient broth (NB) purchased from Sigma Aldich. Two myoviridae bacteriophages specific to strain DSM291 (DSM100069 and DSM100071, hereafter referred to as P1 and P2), were also purchased from DSMZ. Both bacteriophages were propagated, purified and counted as described previously (Bonilla et al. 2016). After propagation the number of plaque forming units (PFU) in the lysates was established by a PFU counting assay with the DSM291 strain as host. Lysates were stored at 4°C. Incubation temperature was 26°C. *Pythium ultimum*, which has been used in previous studies in bacteriophage-FHs dispersal (You et al. 2022), was inoculated in M9 mineral liquid medium (Sigma Aldrich) and incubated at room temperature under agitation (Lab Shaker, Adolf Kühner AG) at 120 rpm for 5 days. The mycelium was fragmented in a 50-ml Falcon tube (Corning) using an ULTRA-TURRAX® (IKA® T18 basic) at max speed for 10 s, and then washed three times with physiological water (0.9 g l^−1^ NaCl). Hyphal fragment density was assessed with a Neubauer chamber (BIOSYSTEMS® 0.01 mm).

### Experimental systems

Two experimental devices (called hereafter: ‘bacterial trail’ and ‘bacterial bridge’; Figure S1, Supporting Information) were used to evaluate diffusion and active transport of bacteriophages in model abiotic networks. The devices were 3D-printed with a heat-resistant hydrophilic material (3DM-HTR140 resin), and the design, validation, and instructions for their production are described in detail in a previous publication (Kuhn et al. 2022). The liquid film in the ‘bacterial trail’ was created by adding 800 µl of NB in all the wells. In this device, the thickness of the liquid film formed is in the magnitude of 1 mm, which has been shown to result in a hydraulic flow that facilitates diffusion of nonmotile particles, including nonmotile bacterial cells (Kuhn et al. 2022). In the second device (‘bacterial bridge’), the liquid film was established at the horizontal top of the device, where two vertical columns allowed a connection by capillarity. The ‘bacterial bridge’ devices were prepared by first filling the capillaries of the columns with NB (with the device upside down). Then, the device was placed upright between two wells in a 24-well cell culture plate (Costar® 24, Corning) filled with 2.75 ml of NB. Finally, in order to establish the liquid film, the top of the capillaries was connected by adding extra medium on the horizontal part, on each side of the central column. The biotic networks were created using ‘fungal drops’ (Buffi et al. 2023), which consist of media droplets placed at a fixed distance on a transparent surface to enable direct visualization (Figure S1, Supporting Information). FHs of *P. ultimum* were grown by inoculating drops of mycelial suspension (30 mycelial particles µl^−1^) into a Petri dish treated for cell culture (100 mm × 20 mm Cell Culture Treated; Corning). In each Petri dish, six pairs of 15 µl drops were deposited at a distance of 0.8 cm, each pair was separated by 1.5 cm. The plates were incubated at room temperature (25°C) and with 70% relative humidity to avoid evaporation. After 4–5 days, the paired drops were connected by *P. ultimum* mycelia. The connected drops were observed under a stereoscope (NIKON SMZ18) or an inverted microscope (EVOS FL, EVOS M5000, Invitrogen) in order to count the number of connecting hyphae.

### Bacteriophage diffusion in an abiotic system

To determine if the bacteriophages diffuse through the liquid film generated on the ‘bacterial trail’ device, the initial and final PFUs in the inoculation and end wells were compared after 20 h. The devices were prepared as indicated above. After that, 10 µl of a concentrated bacteriophage solution was added to the inoculation well (PFU absolute numbers P1 = 4.2 × 10^6^ PFU; P2 = 7.4 × 10^6^ PFU). Six independent replicates per bacteriophage strain were performed. The ‘bacterial trail’ devices were incubated under sterile conditions for 20 h. Then, 150 µl were sampled from the end wells avoiding aspirating liquid from the trail. Bacteriophage concentration was measured as described previously (Bonilla et al. 2016), but given the small volume, only the centrifugation and chloroform purification steps were performed (no filtration). The lysate plate assays were performed on 6 mm Petri dishes adjusting the volume accordingly to half the volume of a regular assay. Only a 1:10 dilution was performed from the purified lysates. PFUs were recorded after overnight incubation at 26°C. The absolute number of bacteriophages was estimated by calculating PFUs based on the sample volume (150 µl).

### Contribution of mycelia to bacteriophage dissemination

To assess bacteriophage dissemination on biotic networks, the initial and final bacteriophage concentration was compared between two drops connected by *P. ultimum* mycelia. One of the drops was inoculated with 10 µl of bacteriophage lysate. After 20 h, the connected drops were sampled. For each replicate, 6 drops of 15 µl were pooled in 810 µl of physiological water (dilution 1:10). Six independent replicates were performed for each bacteriophage strain and each hyphal category. Six controls without *P. ultimum* FHs were performed. The bacteriophage clean-up and lysate plate assay procedures were the same as described above. The absolute number of dispersed bacteriophages was estimated by calculating the PFUs based on the sample volume (15 µl).

### Impact of host and nonhost carriers in active transport of bacteriophages

The ‘bacterial bridge’ device was used to determine the effect of active transport by motile bacteria (host or nonhost carriers) in an abiotic system. The initial and final bacteriophage concentrations were compared after inoculating the start well with 100 µl of bacteriophage lysate (1.3 or 6.4 × 10^8^ PFU) in the presence of the respective bacterial carrier (DSM291 as host or KT2440 as a nonhost) at an optical density of 0.7–0.8. For each carrier and bacteriophage strain, six independent replicates were performed. The devices were maintained under sterile conditions for 20 h. Then, 1.5 ml was sampled from the end well. The samples were then cleaned up and plated as described before (dilutions 1:1 to 1:10^8^). The absolute number of transported bacteriophages was estimated by calculating the PFUs according to the sampled volume (1.5 ml). In order to assess the effect of carriers in FH, 10 µl of the respective bacterial inoculum was coinoculated into the same drop with the bacteriophages (3.6 × 10^6^ PFU). After 20 h, each final drop was sampled (15 µl) and mixed into 1.485 ml of physiological water (1:10^2^ dilution), and quantified as indicated above.

### Statistical analysis

To assess whether the biotic network has a significant effect on the dispersal efficiency of bacteriophages, a linear model was generated by including the dissemination efficiency of bacteriophages with respect to the system (abiotic vs biotic). The bacteriophage strain (P1 vs. P2) was added to the model as a covariate since the strain and the interactions between the experimental system and the strain were not significant (in the full model). We performed a Two-Way ANOVA analysis of this adjusted model. Each comparative group comprised 12 values. To determine the impact of host and nonhost bacterial carriers on bacteriophage transport, a linear mixed-effect model was created to explain the variation in phage dissemination efficiency. In this model, phage dissemination efficiency was used as the response variable. We included the experimental system (bacterial bridge vs. fungal hyphae), the carrier bacterium (host vs. nonhost), and the bacteriophage strain (P1 vs. P2) as fixed effects. We then performed a Two-Way ANOVA analysis with the full model. Each comparative group comprised 12 values. All analyses were carried out within the R statistical environment (Team 2021).

## Results

### Dissemination of bacteriophages along abiotic and biotic dispersal networks in the absence of carrier bacteria

**Figure 1.**
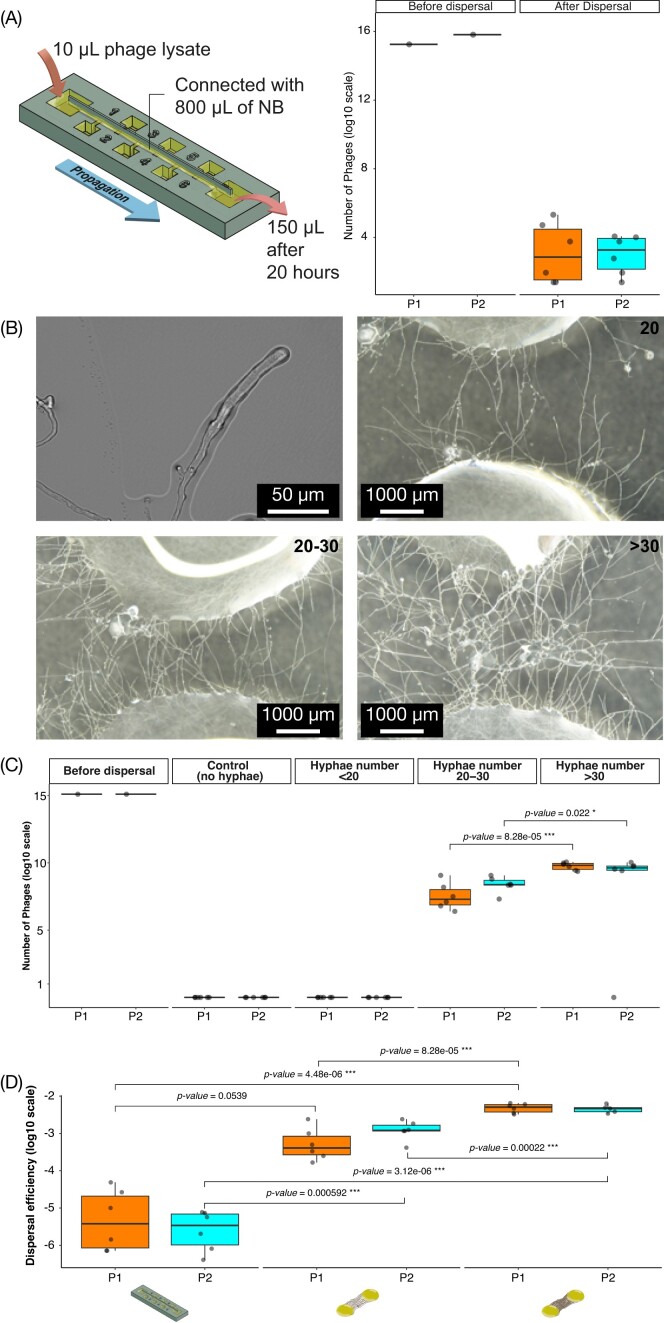
Bacteriophage dissemination in liquid films formed on abiotic or biotic networks. (A) Dissemination on the abiotic system was measured using the ‘bacterial trail device’ (left). The number of bacteriophages propagated to the end well was measured 20 h after inoculation in the start well. Bacteriophage numbers were quantified by PFU. (B) Dissemination on biotic networks was measured on FHs formed by the hyphae of *P. ultimum* using the drop system after the same incubation time. Drops were connected by different number of hyphae: below 20, between 20 and 30, and above 30 hyphae as illustrated in the images. (C) The number of bacteriophages was quantified before (left columns) and after 20 h for the control (no hyphae), below 20, between 20 and 30, and above 30 hyphae. (D) Box plots indicating the dissemination efficiency (ε, calculated as the ratio of final over initial number of bacteriophages). Only the categories in which dissemination was measured were included. All the experiments were run in six independent replicates. The raw data are provided in Table S3 (Supporting Information). The statistical significance of comparisons between treatments are shown on the box plots. The sketch in (A) was modified from Kuhn et al. (2022).

In order to measure the efficiency of bacteriophage dissemination without multiplication along abiotic and biotic networks, we first evaluated their diffusion in liquid films on abiotic and biotic (mycelia of *P. ultimum*) surfaces. Diffusion along abiotic hydrophilic surfaces was studied in the 3D-printed ‘bacterial trail’ device; Fig. 1A). Both bacteriophages, P1 and P2 were found to disseminate through the liquid films genereated with this device (Fig. 1A). To establish the efficiency of bacteriophage dissemination (ε), the ratio between the number of viral particles in the inoculation well and the number detected in the end-well was calculated after 20 h of incubation. The efficiency of dissemination of bacteriophages in the ‘bacterial trail device’ (ε*_trail_*) was calculated to be 1.47 × 10^−5^ for P1 and 4.00 × 10^−6^ for P2, meaning that only a handful of bacteriophage particles in a million disseminated through the liquid film. Next, we evaluated the efficiency of dissemination of bacteriophages across the liquid films formed on FHs formed by the mycelia of *P. ultimum* (ε*_FH_*). In FHs, the number of hyphae connecting two drops could not be controlled and was variable between replicates. In addition, the exact number of hyphae crossing adjacent drops could not be precisely quantified when the drops were connected by more than 30 hyphae (due to multiple overlapping hyphae). Therefore, the number of connecting hyphae was estimated as a range with three categories: drops connected by less than 20 hyphae, connected by 20–30 hyphae, and connected by over 30 hyphae (Fig. 1B). The two bacteriophage strains disseminated along FHs only when drops were connected by 20–30 or over 30 hyphae. The ε*_FH_* was calculated as the ratio between the number of viral particles in the inoculation drop and the number detected in the connected drop after 20 h. The ε*_FH_* increased one order of magnitude with an increasing number of connecting hyphae for both bacteriophages (Fig. 1C). Even though bacteriophage dissemination on mycelia was still limited, ε*_FH_* was higher by one to three orders of magnitude than ε*_trail_* (Fig. 1D). This difference was statistically significant when comparing the two dispersal networks (abiotic vs. biotic), but not when comparing the two bacteriophage strains (Table S1, Supporting Information). A summary of the dissemination efficiency under different treatments is present in Table S2 (Supporting Information).

### Impact of host and nonhost bacterial carriers on bacteriophage dissemination along dispersal networks

**Figure 2.**
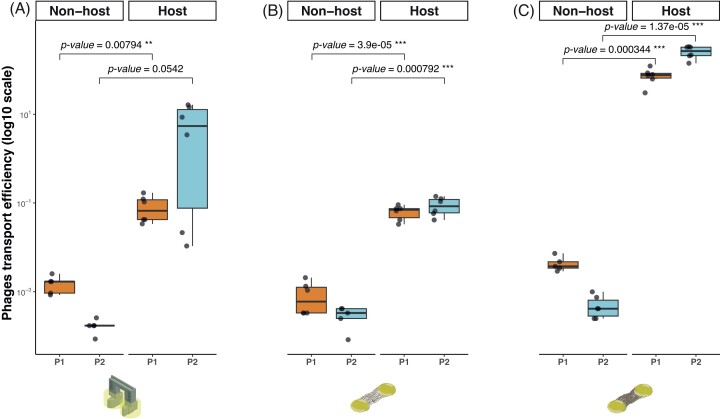
Effect of host (*P. putida* DSM291) and nonhost (*P. putida* KT2440) bacterial carriers on dissemination efficiency. (A) Bacteriophage dissemination efficiency associated to transport in the presence of a bacterial carrier on an abiotic network (‘bacterial bridge’ device). (B) and (C) Bacteriophage dissemination efficiency in biotic networks formed by *P. ultimum* FHs. As in the case of Fig. 1, the number of connecting hyphae was categorized as (B) 20–30 or over 30 (C) hyphae. All the experiments were run in six independent replicates. The raw data is provided in Table S6 (Supporting Information). The statistical significance of comparisons between treatments are shown on the box plots. The sketch in (A) was modified from Kuhn et al. (2022).

We next measured the effect of host and nonhost bacteria as carriers. In this case, dissemination includes not only the transport, but also the possibility for multiplication of the bacteriophage within the host carrier. To reduce the potential for dissemination in the absence of carriers, the ‘bacterial bridge’ device was used. In this device, active swimming has been shown to be required for bacterial dispersal (Kuhn et al. 2022). Accordingly, in the absence of a bacterial carrier, no dissemination of bacteriophages was found. The dissemination efficiency of bacteriophages transported by the two motile bacterial carriers (ε*_Bridge_*) corresponded to 3.63 and 7.13 × 10^−4^, for the host (strain DSM291) and nonhost (strain KT2440), respectively. Thus, the host bacteria were 5.04 × 10^4^ times more efficient for dissemination (combined effect of transport and multiplication) of bacteriophages than the nonhost carrier bacteria (Fig. 2). The same experiments were then performed using the mycelia of *P. ultimum*. The dissemination of bacteriophages in FHs in the presence of a nonhost bacterial carrier (ε*_FHcarrier_*) did not vary significantly across the experimental systems for the two bacteriophage strains. In contrast, the host carrier enhanced dissemination across the experimental systems. Moreover, ε*_FHcarrier_* was the most efficient between drops connected by over 30 hyphae. The differences between networks (abiotic/FHs), carriers (host/nonhost), bacteriophage strains (P1/P2), and the combinations between those factors on the ε of bacteriophage were all statistically significant (Table S4, Supporting Information). A summary of the dissemination efficiency in the presence of host and nonhost carriers under different treatments is present in Table S5 (Supporting Information).

## Discussion

Our study determined the effect of abiotic and biotic dispersal networks on the dissemination of bacteriophages in the presence and absence of host or nonhost carrier bacteria. The factorial design varying the type of network and bacterial carriers one at a time, enabled us to demonstrate the importance not only of diffusion, but also of transport by bacterial carriers and multiplication within the host, in the dissemination of bacteriophages on biotic dispersal networks formed by mycelia (FHs) in the presence of bacterial host carriers.

Dissemination in the absence of carrier bacteria: despite of a very low bacteriophage dissemination efficiency in the ‘bacterial trail device’, we found bacteriophage dissemination in both the abiotic and the FHs systems over a distance of 40 mm after 20 h (Fig. 1). Diffusion in the ‘bacterial trail device’ was one to three orders of magnitude lower than in FHs. However, bacteriophage dissemination in FHs only occurred in the presence of over 20 hyphal connections (Fig. 1). Although a direct comparison between the two systems is difficult as the ‘bacterial trail’ device may provide different connectivity and physicochemical conditions than hyphal surfaces (e.g. due to the secretion of hyphal surfactants), our results suggest that bacteriophage dispersal on hyphal surfaces may take place (Fig. 1). Observing bacteriophage dissemination in the presence of high hyphal numbers suggests that bundling neighbouring hyphae may have caused a volume increase of liquid paths, thereby facilitating phage dissemination. It also may explain the difference to results found by You et al. (2022), who did not find bacteriophage dispersal along *P. ultimum* hyphae in the absence of bacterial carriers. You et al. (2022), applied an *Escherichia* T4 virus to an existing *P. ultimum* network already bridging an air gap between agar patches. Although the reasons for the observed difference may be multifaceted (e.g. bacteriophage size, bacteriophage load, travel time, distance, and so on), we propose here that a combination of hyphal abundance and secondary hyphal growth may have promoted the dissemination of bacteriophages P1 and P2 in this study. In contrast to static abiotic networks, the active growth of FHs can influence dissemination. For instance, tips of growing hyphae are considered to be more hydrophilic than older hyphae and, hence, allow for broader liquid films and better diffusive dissemination of bacteriophages (Kohlmeier et al. 2005).

Dissemination in the presence of carrier bacteria: for the first time, we quantify the effect of closely related host and nonhost bacterial carriers on bacteriophage dissemination. The transport of bacteriophages by the host and nonhost carriers greatly enhanced the dissemination in both types of systems investigated (Fig. 2). Previous studies of bacteriophage dispersal associated with bacterial carriers have used nonhost carriers to assess the impact of transport on the encounter of bacteriophages with their host (Yu et al. 2021, You et al. 2022). Another study demonstrated that this mechanism of bacteriophage dispersal is widespread and involves a large range of nonhost bacteria, suggesting that carrier bacteria-facilitated bacteriophage transportation can be a highly relevant process in soil ecology (You et al. 2022). The rationale for choosing closely related carrier strains is that they are likely to share similar ecological niches and provide similar sets of potential receptors for effective bacteriophage attachment during transport. We found that the dissemination efficiency was indeed higher upon transport by the host, regardless of the network used (Fig. 2).

In our experiments with the nonhost carrier, the dissemination efficiency was constant regardless of the network or the number of connections in the biotic network (Fig. 2). This suggests that dissemination by transport in the absence of multiplication is likely limited by a fixed variable such as the number of binding sites on the carrier cells or transport by turbulence created by the dispersal of motile bacteria. A ratio above 1 in the dissemination efficiency by the host signals a positive role for multiplication in addition to transport. The multiplication and dissemination by a population of host carriers probably did not occur simultaneously (or in a synchronized manner in the entire population), as upon burst and release of new viral particles, lysed carriers can no longer contribute to transport, but contribute positively to the number of viral particles that can be transported by other host carriers (or other bacteria in nature) in the dissemination network. Diverse infection strategies in which the bacteriophage does not decimate the population of host cells, including phenomena such as pseudolysogeny, carrier state, or chronic infections (Mantynen et al. 2021), could also contribute towards the positive effect of host carriers on bacteriophage dissemination. Among these mechanisms, pseudolysogeny does not appear to be likely, as this process results in the injection of the genetic material, but not the active multiplication of viral particles (Ripp and Miller 1997, Los and Wegrzyn 2012). In this case, the dissemination efficacy between the nonhost and host carriers would be expected to be equivalent, which was not the case here (Fig. 2). The other two processes are more likely to contribute to the increase in dissemination efficiency by the host carrier. The establishment of a stable equilibrium in the bacteriophage–host populations with some hosts temporarily resistant to lysis (carrier state; Siringan et al. 2014), releasing viral particles without cell lysis (chronic infection; Hoffmann Berling and Maze 1964), or simply dispersing before the cell energy is diverted into bacteriophage multiplication, can all contribute to the increase in dissemination efficiency resulting from transport and multiplication in the host carrier.

Our experimental systems demonstrate both the dissemination of bacteriophages on mycelia and the positive effect of host carriers. However, they also have limitations that should be acknowledged. For instance, the effect of hydraulic flow, which has been shown to occur in the relatively thick (1.35 ± 0.32 mm) liquid films formed on the ‘bacterial trail’ device (Kuhn et al. 2022), might also occur in the mycelial network and contribute to explaining the positive effect of increasing FH connectivity on bacteriophage dissemination. However, the exact thickness of the liquid film on mycelium and the existence of hydraulic flow across FHs still need to be measured. Likewise, the comparison of bacterial movement across the ‘bacterial bridge’ with FHs in a fixed timeframe does not take into account other factors that might affect bacterial movement in FHs, such as chemotaxis, surfactants, or nutrient provision. These factors should be considered in future studies, for instance, by using fungal exudates as the medium for bacterial dispersal. Moreover, in the future, it will be important to develop experimental approaches to measure the effect of alternative bacteriophage infection strategies (i.e. carrier state or chronic infections) to assess the positive effect of the host carrier on bacteriophage transport.

Our results can be considered under a common framework used for other pathogen–host systems in which the dispersal capacity of the parasite is key for understanding of its access to the host (Johnson et al. 2019). One such framework is the so-called BAM model in which the pathogen niche is determined by biotic factors (B; e.g. host availability), abiotic factors (A; e.g. environmental conditions limiting the survival of the pathogen), and the movement capacity (M; e.g. vector-borne) of the pathogen (Escobar and Craft 2016). The same elements have been shown here and in previous studies to be relevant for the study of bacteriophage–host dynamics in unsaturated environments. The use of FHs as dispersal networks can be an innovative element to expand the BAM framework, as dispersal networks formed by fungi and fungal-like organisms affect the dynamics of the carriers (host and nonhost) actively. In this way, future experiments can investigate disease incidence under different dispersal-limited regimes (e.g. heterogeneous distribution of host and nonhost populations connected by biotic/abiotic networks). They will complement previous studies on the role of bacteriophage transport in the context of invasiveness and range expansion of nonhost carriers (You et al. 2022). We expect such future work to offer a more comprehensive understanding of the mechanisms of bacteriophage dispersal in soils (Pratama and van Elsas 2018) and other spatially structured habitats (e.g. fermented foods, sediments, and surface tissues of plants and animals) in which bacteria, fungi, and bacteriophages actively interact and coexist.

## Supplementary Material

uqae004_Supplemental_Files

## Data Availability

The data underlying this article are available in the article and in its online Supplementary Material.
