# Supplementary material for: Host and nonhost bacteria support bacteriophage dissemination along mycelia and abiotic dispersal networks
Source: Microlife. 2024 Feb 20;5:uqae004. doi: 10.1093/femsml/uqae004 (PMC10924533; doi:10.1093/femsml/uqae004)
Supplement: uqae004_Supplemental_Files [file uqae004_supplemental_files.zip › Phage_MS_microLife_SI_revised.docx]

**Host and non-host bacteria support bacteriophage dissemination along mycelia and abiotic dispersal networks**

Running title (50 characters): Bacteriophage dispersal in biotic and abiotic networks

Claire Périat^1^, Thierry Kuhn^1,2^, Matteo Buffi^1^, Andrea Corona-Ramirez^1^, Mathilda Fatton^1^, Guillaume Cailleau^1^, Patrick S. Chain^3^, Claire E. Stanley^4^, Lukas Y. Wick^5^, Saskia Bindschedler^1^, Diego Gonzalez^1^, Xiang-Yi Li Richter^1,2,6^*, Pilar Junier^1^*

^1^Laboratory of Microbiology, University of Neuchâtel; ^2^Laboratory of Eco-ethology, University of Neuchâtel; ^3^National Laboratory, Los Alamos; ^4^Department of Bioengineering, Imperial College London; ^5^Helmholtz Centre for Environmental Research UFZ, Leipzig; ^6^Institute of Ecology and Evolution, University of Bern

*Co-corresponding authors: [li@evolbio.mpg.de](mailto:li@evolbio.mpg.de); [pilar.junier@unine.ch](mailto:pilar.junier@unine.ch)

Running title (50 characters): Dissemination of bacteriophages in unsaturated environments

Keywords (6): Bacterial-fungal-bacteriophage interactions, infection, multiplication, fungal highways,

Journal FEMS µlife

Once Sentence Summary: Demonstration of the positive role of host carriers and fungal highways in the dissemination of bacteriophages.

**Supplementary Figure 1.** Experimental devices used in this study. The ‘bacterial trail’ (a) and ‘bacterial bridge’ (b) devices were 3D-printed with a heat-resistant hydrophilic material [26] to represent model abiotic dispersal networks. The fungal ‘fungal drops’ system (c) [27] was used to generate biotic dispersal networks constitute by hyphae of *P. ultimum* connecting media droplets placed at a fixed distance on a transparent surface.

**Table S1.** Two Ways ANOVA tests of unassisted bacteriophage dispersal in the abiotic (trail) and biotic (fungal highways) systems. The resulting P-values and significance levels are indicated. The difference between the two experimental systems was statistically significant, while the comparison between the two phage strains was not.

| **Variable** | ***p*-value** | **S** *†* |
| --- | --- | --- |
| Experimental system | 1.82×10^-14^ | *** |
| Phage strain | 6.86×10^-1^ |  |
| *†* Signif. code: 0 ‘***’ 0.001 ‘**’ 0.01 ‘*’ 0.05 ‘.’ 0.1 ‘ ’ 1 | | |

**Table S2.** Dispersal efficiency of P1 and P2 under different experimental conditions. The dispersal efficiency was calculated as the ratio between the number of viral particles in the inoculation well/drop and the number detected in the end-well/drop at the end of corresponding experiments. All the experiments were run in 6 independent replicates.

**Table S4.** Two Ways ANOVA tests of the transport efficiency of bacteriophages influenced by the abiotic (“bridge” device) and biotic (fungal highways, drop system) systems, the carrier (host and non-host), bacteriophage strain (P1 versus P2), and their interactions. The resulting P-values and significance levels are indicated. All comparisons were statistically significant.

| **Variable** | ***p*-value** | **S** *†* |
| --- | --- | --- |
| System  Carrier  Strain  System:Carrier  System:Strain  Carrier:Strain  System:Carrier:Strain | < 2×10^-16^  7.52×10^-13^  7.06×10^-6^  < 2×10^-16^  1.59×10^-8^  3.96×10^-6^  5.40×10^-8^ | ***  ***  ***  ***  ***  ***  *** |
| *†* Signif. codes: 0 ‘***’ 0.001 ‘**’ 0.01 ‘*’ 0.05 ‘.’ 0.1 ‘ ’ 1 | | |

**Table S5.** Dispersal efficiency of P1 and P2 under different experimental conditions in the presence of the host (*P. putida* DSM291) and non-host (*P. putida* KT2440) carriers. The dispersal efficiency was calculated as the ratio between the number of viral particles in the inoculation well/drop and the number detected in the end-well/drop at the end of corresponding experiments. All the experiments were run in 6 independent replicates.
