# Supplementary material for: Host and nonhost bacteria support bacteriophage dissemination along mycelia and abiotic dispersal networks
Source: Microlife. 2024 Feb 20;5:uqae004. doi: 10.1093/femsml/uqae004 (PMC10924533; doi:10.1093/femsml/uqae004)
Supplement: uqae004_Supplemental_Files [file uqae004_supplemental_files.zip › Springer permissions.docx]

**From:** Open Research Support <orsupport@springernature.com>
**Date:** Saturday, 10 February 2024 at 16:56
**To:** JUNIER Pilar Eugenia <pilar.junier@unine.ch>
**Cc:** CAILLEAU Guillaume <guillaume.cailleau@unine.ch>
**Subject:** Re: Enquiry copyright - Ticket ID [#8628781]

E-MAIL EXTERNE ¦ Attention aux liens et pièces jointes

Dear Dr. Junier,

Thank you for contacting Springer Nature.

 Copyright on any research article in open access (OA) journals published by Springer Nature is retained by the author(s). Authors grant Springer Nature a license to publish the article and identify itself as the original publisher. Authors also grant any third party the right to use the article freely as long as its integrity is maintained and its original authors, citation details and publisher are identified.

OA articles in Springer Nature journals are predominantly published under Creative Commons Attribution v4.0 International licence (CC BY). For more information please read our [journal policy](https://www.springernature.com/de/open-research/policies/journal-policies). 

If you have any questions, please do not hesitate to contact us quoting your Ticket ID [#8628781].

 With kind regards,
-- **Conilyn Llander Llander Ocampo**
Global Open Research Support Specialist
Publishing Support
 **Springer Nature Group**
www.springernature.com
